# Patients’ experience of using online interventions for the management of late effects following completion of cancer treatment—protocol for a qualitative systematic review

**DOI:** 10.1186/s13643-026-03194-x

**Published:** 2026-07-28

**Authors:** Ashleigh Ward, Ramsay Lochhead Devaraj, Kate Law, Claire Higham, Janelle Yorke, Daniel Livesey, Sally Taylor

**Affiliations:** 1 Christie Patient Centred Research, The Christie NHS Foundation Trust, Manchester, UK; 2 The Christie NHS Foundation Trust, Manchester, UK; 3 Department of Endocrinology, The Christie NHS Foundation Trust, Manchester, UK; 4 School of Nursing, The Hong Kong Polytechnic University, Kowloon, Hong Kong, China; 5 Division of Nursing, Midwifery and Social Work, School of Health Science, Faculty of Biology, Medicine and Health, University of Manchester, Manchester, UK; 6 The Christie Library, School of Oncology, The Christie NHS Foundation Trust, Manchester, UK

**Keywords:** Online interventions, Post-treatment, Long-term side effects, Late effects, Cancer treatment, Oncology, Qualitative, Thematic synthesis, Systematic review

## Abstract

**Background:**

Global cancer cases are predicted to rise to 35 million by 2050 and survival rates are also increasing, meaning the number of patients experiencing late effects from their cancer treatment is expected to rise. Post-treatment side effects can present physically, psychologically, and functionally. These complications can include peripheral neuropathies, fatigue, cognitive or sexual difficulties, and heightened anxiety or depression.

A wide range of online interventions have been developed as treatment and support tools for cancer patients following treatment, including self-directed or therapy-assisted programs, symptom-reporting applications and education tools. Research to examine the effectiveness of online interventions for the management of late effects has shown these interventions to have a positive impact on outcomes such as managing fatigue, depression, and overall quality of life.

The aim of this study is to systematically identify, review and synthesise the qualitative experiences of patients with cancer using online interventions for managing late effects post-cancer treatment in order to further inform the design and implementation.

**Methods:**

A comprehensive search strategy has been developed to identify studies across six different databases (MEDLINE, EMBASE, EMCARE, PSYCHINFO, CINAHL and The Cochrane Library). Studies will be screened and selected by two independent reviewers. The inclusion criterion includes peer-reviewed qualitative or mixed-methods studies, published on any date, with patients over the age of 18, in any geographical location or setting, and written in English. A Preferred Reporting Items for Systematic Reviews and Meta-Analyses (PRISMA) diagram will be developed to show the screening and selection process. Once included studies have been identified, Thomas and Harden’s (2008) three stages of thematic synthesis will be followed by two reviewers to generate analytical themes.

**Discussion:**

By reviewing patients’ experience of using online interventions to help with late effects of cancer and its treatment, we hope to be able to provide some guidance for those developing and implementing these interventions into care, to maximise the benefit for patients.

**Systematic review registration:**

PROSPERO (Reference CRD42025619614).

## Background

Cancer, while having a significant impact on individuals on a personal level, also has substantial societal and macroeconomic costs that vary depending on the type of cancer, geographical location, and gender [1]. It was estimated that in 2022 almost 20 million people globally were newly diagnosed with cancer, and the number of new cancer cases is predicted to rise to 35 million by 2050 [1, 2].

While cancer survival rates have increased over the past decade due to new cancer treatments [3], a considerable number of cancer survivors will experience post-treatment side effects often referred to as late effects or long-term side effects [4]. These can stem from both cancer and its treatment and cover a wide range of physical and psychosocial changes [4] and can have a sustained impact on patients’ quality of life [5]. Specific examples of late effects can include pain; fatigue; cognitive impairment; sexual function difficulties; fear of recurrence; and elevated anxiety or depression [6, 7].

Managing the transition from being on active treatment to active follow-up involves equipping patients to self-manage late effects. This relies on adequate provision of information and support, which patients have highlighted to be lacking [8]. As a result, side effects that arise post-treatment are often ineffectively managed [9–11]. Research suggests that this is due to oncology practice focusing resources on management of the disease, treatment, and detecting recurrence [11], as well as increased strain on resources due to prolonged survival of cancer patients and the increasing costs of cancer treatment [12].

These difficulties could be alleviated through online interventions that have become a further developed support tool for patients with chronic diseases including cancer [13]. Online and digital interventions are tools that are often developed in the form of applications (apps) and websites that can be accessed by patients or carers via smartphones, tablets and computers either in their own home or in clinical settings. These interventions can include self-directed or therapy-assisted programs aimed at enhancing knowledge and providing support or treatment [14]. Digitally supported self-management tools serve to enhance patient experience, and the use is advocated as part of the national agenda to achieve world class cancer outcomes in the UK [15].

Systematic reviews have been conducted analysing quantitative data assessing the efficacy and acceptability of online interventions. One systematic review of 37 studies exploring specific outcomes (fatigue, depression, anxiety, and overall quality of life) in cancer survivors found online interventions to have a positive impact [16]. This review recommended the incorporation of information technologies in the development of intervention strategies to better support patients with their self-management. Despite this, there is no standard approach to post-treatment services, no guidance on which interventions should be implemented, and access to digital support is still not equitable [17].

Future research on patient-centred oncology care post-treatment should focus on informing appropriate services on how best to meet the individual needs of people following cancer treatment and making this information available and accessible [18–21]. While quantitative research is important in identifying patterns and trends [22], qualitative research can provide a comprehensive understanding of patients’ experiences as well as the delivery of oncology care and interventions [23, 24]. Cancer treatment side effects have been shown to be a significant barrier to adherence to online interventions [25]. Therefore, gaining insight into patient experience would allow a deeper understanding of how best to support patients to overcome this and other barriers, as well as helping to identify strategies to improve engagement with current interventions [26]. This in turn will improve patient satisfaction, another factor impacting patient adherence [27].

Preliminary searches identified sufficient primary qualitative studies to enable meaningful synthesis and interpretation of findings. A systematic review approach was therefore selected to allow for transparent study selection, critical appraisal, and rigorous qualitative synthesis. Our paper aims to provide much needed insight into patients’ experience, and we aim to use the findings from this review to better inform the development and implementation of future interventions for the management of late effects in cancer survivors.

## Methods

### Aim and objectives

#### Aim

To systematically identify, review and synthesise the experiences of patients with cancer using online interventions for managing late effects post-treatment.

#### Objectives

1. Identify and characterise the types of online interventions used by patients with cancer for managing late effects post-treatment.
2. Synthesise the patient-reported experiences of using these online interventions.
3. Provide recommendations for the development and implementation of online interventions based on patient feedback and identified gaps in current practice.

### Inclusion and exclusion criteria (Table 1)

**Table 1:** PCC inclusion and exclusion table

Population	Include: patients with any cancer diagnosis who are post-systemic anti-cancer therapy and/or radiotherapy and/or Surgery and over the age of 18 Exclude: patients with cancer who are still in treatment and are under the age of 18
Concept	Include: patients’ experience of using an online intervention to manage late effects after completing treatment for cancer Exclude: patients who do not have experience of using online interventions or have experience of using interventions that are not web based or digital
Context	Include: studies using qualitative or mixed methods design, in any geographical location or setting, published on any date in English language Exclude: grey literature (e.g. thesis or commentaries)

### Search strategy

A comprehensive search strategy has been developed (Table 2) with support from clinical experts and an experienced librarian. These search terms will be used on the following six databases: MEDLINE, EMBASE, EMCARE, PSYCHINFO, CINAHL and The Cochrane Library. The results will be merged and duplicates removed using a reference manager. A limit has been applied to the search strategy to only include publications in English, we are currently unable to translate studies published in other languages. This restriction has shown to have little effect on the conclusions of systematic reviews [28]. The protocol is reported using Preferred Reporting Items for Systematic reviews and Meta-Analyses Protocol (PRISMA-P) guidelines and is registered on the International Prospective Register of Systematic Reviews (PROSPERO) (Reference CRD42025619614).

**Table 2:** Full search strategy implemented on Ovid MEDLINE

Ovid MEDLINE(R) ALL < 1946 to February 17, 2025 >		
**# ▲**	**Searches**	Results
1	(Web* or Tech* or Platform* or digital* or E-learning* or E-advice* or mHealth* or eHealth* or ICT or Network* or Interface or ipad or tablet or app*).ti	1,889,999
2	((Online or web) adj3 (tool* or intervention*)).ti	4409
3	*Internet-based intervention/or *Digital Health/or *Telemedicine/or *Electronic Mail/or *Internet/or *Online Social Networking/or *Online Systems/or *Virtual Reality * exposure Therapy/or *Virtual Reality/or *Therapy, Computer-Assisted/or *Computer/or *telecommunication/or *software/or *Mobile application/	178,264
4	1 or 2 or 3	2,020,470
15	(Cancer or Tumo?r* or Malignan* or neoplas* or oncolog* or metasta* or gliom* or sarcom* or melanom* or leukaem* or leukem* or lymphom* or mesotheliom* or radiotherap* or chemotherap* or chemo* or immunotherap* or brachytherap*).ab,ti	4,878,523
6	exp Neoplasms/	4,075,157
7	5 or 6	5,839,763
8	(Qualitative or grounded theory or interview* or nursing methodology research or focus group* or phenomenolog* or ethnograph* or case stud* or historic research or action research or experience* or view* or perspective* or perception* or attitude* or preference* or satisfaction* or opinion* or insight* or thematic analysis or narrative inquiry or storytelling).ab,ti	4,454,978
9	exp qualitative research/or exp grounded theory/or exp interview/or exp nursing methodology research/or exp focus groups/	170,484
10	8 or 9	4,476,185
11	(Late effect* or Lasting effect* or long term effect* or long-term effect* or After effect* or Permanent side effect* or Permanent symptom* or Lasting side effect* or Lasting symptom* or chronic side effect* or Chronic symptom* or long term side effect* or Long-term side effect* or long term symptom* or Long-term symptom* or post treatment or delayed side effect* or delayed symptom* or Psychological long-term effect* or Psychological long term effect* or psychological late effect*).ab,ti	141,584
12	4 and 7 and 10 and 11	386
13	limit 12 to English language	374

### Study screening and selection

Studies identified through the search strategy will initially be reviewed and any duplicates removed using Rayyan.ai. The abstracts will be independently screened by two reviewers using Rayyan.ai. Abstracts that are potentially eligible at this stage will be marked for full-text review; any studies not eligible at this stage will be excluded. Only peer-reviewed studies will be included to ensure evidence has been subject to peer review and meets the methodological standards required for publication. Including grey literature may weaken the reliability of our findings by including reports that potentially lack information on methodology and have not undergone rigorous scrutiny. Full-text versions of potentially eligible studies will be reviewed independently by both reviewers, and reasons for studies excluded at this stage will be given (e.g., incorrect study design, incorrect population). Conflicts will be resolved through discussion with a third member of the research team. All records will be stored and managed using Rayyan.ai. The references for any studies included in the final review will be screened to identify any further potential studies. The number of studies at each stage of screening will be outlined in a PRISMA diagram.

### Critical appraisal

To ensure reliable conclusions, the validity and reliability of eligible studies will be assessed to contextualise their potential limitations and strengths alongside the findings, and to identify potential methodological limitations. All eligible studies will be evaluated using the Joanna Briggs Institute qualitative critical appraisal tool [29–31]. Mixed methods studies will be appraised using the same tool, however focusing solely on the qualitative methods, data collection and data analysis. The tool consists of 10 items to be completed by two reviewers independently.

Following the appraisal of eligible studies, data synthesis and data analysis, the confidence of review findings will be assessed via the “Confidence in the Evidence from Reviews of Qualitative Research” (GRADE CERQual) approach [32]. The tool allows authors to assess the review findings surrounding patient experience of online interventions to manage late effects through the lens of four components: methodological limitations, relevance, coherence, and adequacy of the data analysed in the included studies.

### Data extraction

A data extraction form will be created to record relevant data enabling comparison across the included studies. Extracted data will include author, title, year of publication and the geographical location. Study characteristics will also be extracted, including study design, aims and objectives, methods of data collection and the method of data analysis. Demographics of participants will be extracted, including gender, age, ethnicity, cancer type and time since treatment completion and time since diagnosis, if this is reported. We will also extract data on the key features of the intervention.

Qualitative data and findings from all included studies will then be extracted into NVivo to undergo thematic synthesis.

### Thematic synthesis

Thematic synthesis will be used for the data analysis of the systematic review due to its flexible, transparent, and systematic methods [33]. Thematic synthesis has been shown in previous qualitative systematic reviews to be suitable for exploring participants’ views of interventions and identifying factors that influence an intervention’s success [34]. The three stages of thematic synthesis set out by Thomas and Harden (2008) will be adhered to [33]. During stage 1, both reviewers will independently code the extracted text line by line according to its meaning and content. Each sentence will have a minimum of one code. Once all text has been coded, reviewers will check all coded text for consistency. Step 2 is the development of descriptive themes; codes developed in stage 1 will be grouped into a tree structure with hierarchical themes. Following this, stage 3 is the process of creating analytical themes. Reviewers will do this independently to start with and then meet with all other authors to discuss and agree on the final analytical themes. The framework will be developed throughout the synthesis via collaboration of both authors.

### Timeline

Searches were completed on the 17th of February 2025 and analysis is currently ongoing. Searches will be rerun to ensure we capture any new studies that have been published in the time we are taking to complete the analysis.

## Discussion

To the best of our knowledge this protocol represents the first systematic review investigating the qualitative experience of patients with cancer using online interventions for the management of late effects. As such, the search strategy for this review may not include every potential search term to find eligible studies. We have tried to overcome this by collaborating with an experienced librarian in designing the search strategy. Thematic synthesis as an analysis tool also has its limitations as its synthesis is subjective to the researchers’ interpretation. Although, it allows for exploration into the individuals’ experience to give context to the clinical evidence as well as insight into patients’ needs and how to meet them. Both reviewers will conduct the analysis independently, with any disputes resolved with a third reviewer. Taking into consideration the heterogeneous nature of eligible studies resulting from the search strategy, we anticipate varying outcome measures collected at different time points as well as varied attrition rates. This may make it difficult to draw firm conclusions. However, this will still allow comparison against existing data to inform health care professionals surrounding how best to support patients. Finally, only including English publications may result in reporting and selection bias, but this will be addressed via the analysis.

This review aims to gain a deeper understanding of patients’ experience with using online interventions for the management of late effects, these might include barriers to adherence, perceived efficacy and suggested improvements. These insights will allow us to provide evidence-based recommendations for the development and implementation for online interventions used to manage late effects. These could be determining the optimal time for introducing interventions to this population of patients, recognising the type and level of support patients might need while using online interventions and highlight features that have a significant benefit to patients. Providing guidance may support the optimisation of online interventions for late effects management, maximise benefit to patients and potentially ease strain and free up resources within the healthcare system.

## Data Availability

The datasets analysed in this study are publicly available.

## Abbreviation

PRISMA: Preferred Reporting Items for Systematic reviews and Meta-Analyses
